# Behavioral and Emotional Disorders in Children and Their Anesthetic Implications

**DOI:** 10.3390/children7120253

**Published:** 2020-11-25

**Authors:** Srijaya K. Reddy, Nina Deutsch

**Affiliations:** 1 Department of Anesthesiology, Division of Pediatric Anesthesiology—Monroe Carell Jr. Children’s Hospital, Vanderbilt University Medical Center, 2200 Children’s Way Suite 3116, Nashville, TN 37232, USA; 2 Division of Anesthesiology, Pain and Perioperative Medicine—Children’s National Hospital, The George Washington University School of Medicine and Health Sciences, 111 Michigan Avenue NW, Washington, DC 20010, USA; ndeutsch@childrensnational.org

**Keywords:** child behavioral disorders, attention deficit and disruptive behavior disorders, perioperative care, anesthesia, autism spectrum disorder, premedication, emergence delirium

## Abstract

While most children have anxiety and fears in the hospital environment, especially prior to having surgery, there are several common behavioral and emotional disorders in children that can pose a challenge in the perioperative setting. These include anxiety, depression, oppositional defiant disorder, conduct disorder, attention deficit hyperactivity disorder, obsessive compulsive disorder, post-traumatic stress disorder, and autism spectrum disorder. The aim of this review article is to provide a brief overview of each disorder, explore the impact on anesthesia and perioperative care, and highlight some management techniques that can be used to facilitate a smooth perioperative course.

## 1. Introduction

Anxiety and fear are common emotions for children to experience when faced with the need to undergo a surgical or diagnostic procedure, with Kain and colleagues determining that up to 60% of all children undergoing anesthesia and surgery report significant anxiety [1]. Several risk factors for perioperative anxiety have been identified. Younger children are more likely to exhibit separation anxiety from parents and are less cooperative on anesthetic induction compared to older children [2]. Situational anxiety of the mother, temperament of the child, and the quality of previous medical encounters also influence the incidence of anxiety [1]. Other studies have found that younger age, non-Anglo ethnicity, and female sex are pre-existing risk factors [3]. Many resources and techniques, including Child Life specialists, parental presence and premedication, are available to help facilitate a safe perioperative experience in the face of significant anxiety and fear. Newer technologies, such as applications on smart devices and virtual reality devices, are also being used more regularly to help distract patients and alleviate their negative emotions.

In patients with known behavioral diagnoses, a tailored approach that takes into account a child’s disorder and best prepares them for the challenges of the perioperative environment is key to a successful and safe surgical experience. In this review article, common behavioral and emotional disorders will be discussed with a focus on their impact on anesthesia and perioperative care. Various management techniques that can be used to facilitate a smooth perioperative course will also be reviewed.

## 2. Disorders

Several behavioral and emotional disorders exist in children and adolescents, which potentially affect the perianesthetic care of these patients. These include anxiety, depression, oppositional defiant disorder, conduct disorder, attention deficit hyperactivity disorder, obsessive compulsive disorder, post-traumatic stress disorder, and autism spectrum disorder. The prevalence, characteristics, and potential perioperative issues these disorders can produce are summarized in Table 1.

### 2.1. Anxiety and Depression

It is not uncommon for children to be diagnosed with depression or anxiety, and many of these children have associated symptoms that require them to engage with the hospital setting. Anxiety is characterized by excessive and uncontrollable worry and fear that results in significant impairment or distress [4]. Anxiety in children can further be classified as separation anxiety, generalized anxiety, social anxiety, panic disorder, or a specific phobia, such as needle phobia. They often present with somatic complaints, ranging from abdominal pain, chest pain, and headaches to shortness of breath, sweating, and tachycardia [4].

The incidence of diagnosis of either anxiety or depression among children aged 6–17 years has increased over the last several years, from 5.4% in 2003 to 8% in 2007 and 8.4% in 2013 [5]. Anxiety disorders are more prevalent in children than attention deficit hyperactivity disorder (ADHD) or mood disorders [6]. Childhood anxiety disorders, especially if left untreated, increase the risk of psychiatric disorders in adulthood, including depression and substance abuse [7]. Children with anxiety disorders often have significant functional impairment that can impact their academic performance, attendance at school, social interactions, and relationships [8]. Thoughts of parental separation, loss of control, pain during or after surgery, an unfamiliar environment, and the uncertainty of anesthesia and surgery can trigger or exacerbate anxiety in children. In younger children, this can manifest as refusal to separate from the parents, crying, thrashing, and verbal resistance [4].

Depression is a persistently sad or irritable mood that affects a child’s behavior at home, in school and with peers. This can include decreased interest in activities, feelings of hopelessness, and suicide [8]. Characteristics of depression are summarized in Table 1, and a diagnosis of depression is usually made when these symptoms last for two weeks or longer, interfering with a child’s ability to function. Depression is a serious health condition, which, if left untreated, can pose a risk of suicide [9]. Some children will present with physical complaints, such as headaches and stomachaches, while others might have increased defiance and declining performance at school. The prevalence of childhood depression increases with increasing age, ranging from 3% under 13 year of age to 8% during adolescence [8]. While depression can affect children of all ages and both genders, girls are much more likely to develop depression during adolescence [8]. It can be caused by chronic medical conditions, stressful or traumatic life events, environmental issues related to family or social problems, alcohol or drug use, physical or sexual abuse, or when there is a family history of depression [8]. There are many other mood disorders that present with depression such as adjustment disorder with depressed mood, seasonal affective disorder, bipolar disorder, and disruptive mood dysregulation disorder; however, exploring each one of these mood disorders is beyond the scope of this review. Unfortunately, in children, depression does not always seem obvious, and many children will insist that they are doing fine or will deny having any problems.

In addition to receiving a premedication for anxiolysis, patients with anxiety and/or depression also benefit from strategies like psychological preparation programs and complimentary therapies such as reading a storybook, age-appropriate teaching interventions, and the use of Child Life specialists [10]. While parental presence at induction works for certain patients, the combination of high anxiety and low sociability in a child with high anxiety in parents is predictive of elevated perioperative anxiety [11]. Distraction with audiovisual aids has also been shown to be effective in decreasing anxiety [10]. Speaking in a quiet, reassuring voice, avoiding a condescending tone, validating the child’s feelings, using age-appropriate language, and avoidance of terminology may help to decrease anxiety levels [12]. Specific interventions are discussed in more detail below. Screening for self-injury or suicidal risk prior to anesthesia or surgery is an important step in the preoperative assessment. If an anxiety disorder or depression is suspected, a referral to a behavioral health specialist for further assessment would be reasonable.

### 2.2. Disruptive Behavior Disorders

#### 2.2.1. Oppositional Defiant Disorder (ODD)

Oppositional defiant disorder (ODD) is diagnosed in children who are uncooperative, defiant, and hostile towards peers, parents, and authority figures, with approximately 3% of children meeting criteria [13]. It occurs more commonly in boys and is often associated with mood or anxiety disorders, conduct disorder, and ADHD [13]. Contributing factors include genetic predisposition, parenting problems, lack of supervision, inconsistent or harsh discipline, abuse, or neglect. Children with ODD can be very difficult to treat in the medical setting. Those who are receiving psychotherapy (individual or family), cognitive problem-solving and/or social skills training, or pharmacologic therapy tend to do better with medical direction. If an adolescent with ODD is very aggressive, defiant, and does not give their assent for surgery, the procedure may have to be postponed [14]. We have a duty to determine the ability and competence of a child to give their assent for anesthesia and surgery [15]. In some countries and jurisdictions, adolescent assent is required by law. Since most children with ODD are competent, it can become an issue of ethics when trying to persuade them to proceed when they clearly do not want to.

#### 2.2.2. Conduct Disorder (CD)

Conduct disorder (CD) is often considered to be a more severe form of ODD and affects approximately 3% of school-aged children [16]. It is characterized by a complete disregard and aggression towards others. This can present in the form of pushing, hitting, and biting in early childhood to bullying, vandalism, cruelty, and violence in adolescent years [16]. Boys who have CD are more likely to display aggressive and destructive behavior, while girls are more likely to embrace deceitful and rule-violating behavior. Associated factors include lack of impulse control, child abuse, family dysfunction, parents with substance abuse issues, and poverty [17].

Managing severe CD with aggressive behaviors can be difficult, and it is often treated with antipsychotic medications such as quetiapine, olanzapine, risperidone, and ariprazole [18]. Potential interactions of quetiapine, alonzipine, and clozapine with anesthesia include hypotension, delayed emergence, and constrictive pericarditis; risperidone can cause prolonged QT interval and serotonin-syndrome when combined with paroxetine; and aripiprazole can cause prolonged QT interval, altered glucose metabolism, body temperature dysregulation, and acute dystonic reactions [19]. Children and adolescents with CD benefit tremendously from premedication for anxiolysis and to decrease the risk of aggression and injury. If an intravenous (IV) catheter is in place preoperatively, IV premedication is usually rapid and can facilitate a smooth transition to the operating room. Many children with CD are on chronic benzodiazepines in addition to their antipsychotic medications. Alternative medications such as dexmedetomidine or ketamine might be more effective in these patients that have built up a tolerance to benzodiazepines [20]. Caution should be used with ketamine as the hallucinogenic effects might not be ideal in children with CD. The difficulty arises when patients with CD resist IV placement or refuse inhalational induction. Similar to ODD, the issue of assent comes into play, and surgery might have to be rescheduled.

### 2.3. Attention Deficit Hyperactivity Disorder (ADHD)

ADHD is characterized by inattention, impulsivity, and hyperactivity. As there is an increasing number of children diagnosed with ADHD, with a prevalence of 2–20% of school-aged children, it is likely that many of these children will present for surgery and anesthesia at some point [21]. Preoperative anxiety, difficulties with induction and emergence, behavior changes in the postoperative period, and medication interactions are concerns for children with ADHD. In a study published in 2010, researchers from the University of Michigan prospectively evaluated 268 children undergoing elective surgery. Half of these patients had ADHD, while the other half did not. What they found was that children with ADHD were less cooperative with induction and had more behavioral issues postoperatively [21]. Specifically, children with ADHD had challenges with concentration and decision-making, were more disobedient, impulsive, fidgety, had a poor appetite, were difficult to engage with, and displayed an increase in temper tantrums following surgery [21]. These findings are important in terms of notifying parents about the possible maldaptive behavioral issues that might occur in children with ADHD after surgery or anesthesia. In addition, it is important to note that methylphenidate, a drug that is often used to treat ADHD, can reduce the effects of midazolam and increase anesthetic induction requirements [22,23]. Another medication that is gaining popularity in the treatment of ADHD is guanfacine, a non-stimulant alpha-2 agonist [24]. Missing a dose of guanfacine can result in withdrawal symptoms, including hypertension and tachycardia, so patients should continue taking this medication even on the day of surgery [24].

### 2.4. Obsessive-Compulsive Disorder (OCD)

Obsessive-compulsive disorder (OCD) is an anxiety condition that afflicts a child with unwanted thoughts, images, or impulses—called obsessions—that are impossible to suppress, causing a great amount of stress and worry. Ritualized actions, or compulsions, alleviate the anxiety caused by these obsessions [25]. The prevalence is now estimated to be 0.25–4% [25]. While most children seek reassurance from their parents and/or authority figures, children with OCD tend to ask repeated questions pertaining to the future [26]. Cognitive behavior therapy and selective serotonin reuptake inhibitors (SSRIs) are the mainstay treatment options for OCD [26]. Deep brain stimulation surgery has been used to treat this disorder in adults with some promising results [27]; however, there is limited data in children. Children with OCD can have extreme preoccupations with dirt and germs and an extreme need to know or remember things that are rather trivial [25]. Excessive attention to detail and extreme worrying about something terrible happening are likely to be magnified prior to having surgery or anesthesia. While no large-scale studies of these patients in the perioperative setting exist, reassurance, clear communication, and adequate anxiolysis will help facilitate a smooth perioperative course for these patients. A calm, supportive environment and demeanor and reasonably supporting the child’s coping mechanisms can help improve outcomes as well.

### 2.5. Post-Traumatic Stress Disorder (PTSD)

All children may experience very stressful events in their lives that can impact how they think and feel. Most children recover from stressful events quickly; however, sometimes children who experience severe stress, such as from an injury, significant medical events, death of a family member or friend, or violence or abuse, could be affected long term [28]. When a child experiences direct trauma or witnesses it happening to someone else, the resultant stress can interfere with their daily activities and relationships. According to the National Center for PTSD, 100% of all children who witness the murder or sexual assault of a parent develop PTSD; 90% of children who are sexually abused develop PTSD; 77% who are injured in, or witness, a school shooting develop PTSD; and 35% of kids in urban areas who are exposed to violence in the community develop PTSD [29]. These horrific experiences make children with PTSD very fragile and distrusting of most people. A growing number of studies have confirmed PTSD in children and adolescents after hospitalization and medical procedures [30,31]. The incidence of medically related PTSD is as high as 12–31% in children undergoing cardiac surgery [32]. Providing developmentally appropriate information and presenting opportunities for them to exercise some degree of control, such as allowing them to select a distracting activity or toy or the position they want to be in when they fall asleep, can mitigate some of their apprehensions [33]. Asking for permission before approaching or touching the child with PTSD is important for their sense of boundaries. Evidence suggests that active distraction techniques like engaging the child in interactive play or in nonprocedural talk are the most effective in reducing stress in children with PTSD [34].

### 2.6. Autism Spectrum Disorder (ASD)

Autism spectrum disorder (ASD) is diagnosed in 1 out of every 100 children. There is a 55% associated with intellectual disability, a 70% association with other disorders, such as ADHD, anxiety, and CD, and a 30% association with epilepsy [34]. The hallmarks of ASD are persistent deficits in social communication, restrictive and repetitive interests, behaviors, and activities, and sensory hypersensitivity [35]. No two children with ASD are exactly alike, with varying degrees to which these symptoms exist. Therefore, ASD is further classified based on the level of support needed into three categories: requiring support (level 1); requiring substantial support (level 2); and requiring very substantial support (level 3) [36]. These characteristics have a significant impact in the perioperative setting. Children with ASD often do not have the ability to comprehend what is going on and have difficulties communicating their thoughts and fears [35]. In a study comparing children and adolescents with low-functioning ASD to matched controls, the ASD patients were more likely to react to blood draws with other-injurious behaviors (OIB) such as slapping or kicking [37]. They might not like the feeling of a hospital gown, the sounds of other children, fluorescent lights, and may not tolerate the bitter taste of certain premedications. This, along with prior bad interactions with healthcare, can lead children with ASD to be agitated, disruptive, and very distrustful of the hospital and all those who work in it. In adolescents, their larger size and strength can make the ability to control aggressive behaviors more difficult.

It is important for us to be sensitive to the parents of children with ASD and receptive to their child’s specific needs. To minimize risks, children with ASD should be identified early and offered pre-hospital and pre-surgical preparation. Many children with ASD are intelligent enough to cooperate as long as they are prepared and understand what is expected [36]. A flexible admission process, minimal preoperative waiting times, and a quiet room for both pre- and postoperative care are ideal [35]. Some simple suggestions to increase the likelihood of a successful outcome are to not force a children with ASD to change into the hospital gown, use distraction techniques to help facilitate separation from parents, minimize loud noises and bright lights, and allow them to have a familiar comfort item with them in the operating room. The use of premedication is strongly recommended for children with ASD. The use of restraint might need to be used as a last resort; however, it is associated with a loss of dignity for patients and psychological and physical harm [35,36]. If restraint is unavoidable, make sure that the parents or caregivers understand and give consent and that trained staff are involved [38]. Many parents of children with ASD understand that temporary restraint might be required just to administer premedication, like intramuscular (IM) ketamine, but it can be discontinued once the patient is adequately sedated [39].

## 3. Perioperative Management

Surgery can be one of the most stressful experiences a child may have, whether or not they have a known behavioral disorder. Approximately 70% of all children demonstrate stress and anxiety before surgery [1]. Various reasons for these behaviors exist, including the child’s concerns of bodily harm, fear of being separated from his/her parents, and loss of control or autonomy [40]. Minimizing anxiety and psychological trauma in the perioperative period has been shown to decrease the incidence of airway complications, emergence agitation, postoperative pain, short- and long-term behavioral changes, patient and family dissatisfaction, and difficult induction with subsequent anesthetics [1,41,42,43,44,45]. Postoperative maladaptive behaviors, such as general anxiety, enuresis, nightmares, and separation anxiety have also been associated with preoperative anxiety [46]. To help ensure a better operative experience for patients and their families, efforts to reduce anxiety should begin in the preoperative setting and continue through induction of anesthesia and into the recovery period.

With an appropriate plan in place, measures can be taken to ensure not only a safe induction but also one that is tailored to the child and limits the stress around this potentially traumatic period. Parents and families should be informed of options that are appropriate regarding anesthetic induction and perioperative care so that they can be a part of the decision-making process to best provide for their child. Parents can help to inform caregivers about the needs for premedication, parental presence, and the type of induction technique (inhalation vs. IV) that has worked well in the past to ensure the smoothest and safest course possible. This planning also includes a backup strategy if the original plan does not work.

For combative children scheduled for elective procedures, postponing the procedure until the child is properly prepared should be considered. However, the majority of children can be well managed in a friendly environment. Allowing them to keep objects that are brought from home with them, such as a favorite toy, blanket, or pacifier can be helpful and provide a sense of security. Introducing them to the anesthesia mask and letting them decorate it with stickers and their favorite flavor can also provide them with a sense of control. In addition to these easy measures, numerous options exist to help to combat perioperative anxiety, whether it is premedication, distraction techniques, or parental presence during induction.

### 3.1. Preoperative Preparation Programs

Preoperative preparation programs, in which patients and families utilize informative resources prior to arrival in the hospital for a procedure, come in many forms. Some examples of these programs include videos of hospital-related material, interactive teaching books, in-person tours, and role-playing with Child Life specialists, and multi-component behavioral preparation programs that teach parents distraction techniques. The utility of these interventions in decreasing perioperative anxiety has been studied, with varying results.

In one study of children aged 2 to 6 years, participants that were randomized to an interactive book exhibited greater anxiety on the day of surgery than those that underwent routine preparation. However, they were less aggressive postoperatively and reported fewer behavioral changes two weeks after surgery compared to the control group [47]. A separate study found that the use of this type of book caused no change in perioperative anxiety compared to a control [48].

Another study looking at the utility of a tour and role-playing with a Child Life specialist found that anxiety was significantly reduced in the preoperative period but not during induction or postoperatively compared to no intervention. The preparation program was associated with increased anxiety in children aged 2 to 3 years, those with emotional lability, and those with previous hospital experience. Participating in the program more than five days prior to surgery was also associated with less anxiety in those older than six years compared to those that participated one day prior to surgery [47]. Similarly, variable results depending on age have occurred with the use of preoperative videotapes of hospital-related materials compared to non-medical videos [2].

ADVANCE (an acronym for Anxiety-reduction, Distraction, Video modeling and education, Adding parents, No excessive reassurance, Coaching, and Exposure/shaping) is a multifaceted program that combines strategies to reduce anxiety with teaching parents distraction techniques, video education before surgery, parental presence at induction of anesthesia (PPIA), and exposure of the child to the anesthesia mask. A study compared ADVANCE to three other groups: PPIA alone; oral midazolam; and a control group. ADVANCE was found to significantly reduce anxiety compared to the other three groups in the preoperative area. There was less anxiety during induction with ADVANCE compared to PPIA and control groups, and it was comparable to the use of midazolam during induction. Emergence delirium and analgesic requirements were less in the ADVANCE group and discharge times were also shorter compared to the other three groups [49]. However, implementation of this type of program is complex and requires significant operational costs.

### 3.2. Child Life

Early identification of children and families who are likely to suffer significant anxiety is important to help develop an effective perioperative plan. In many institutions, patients with known behavioral disorders are identified in the preoperative call to parents. This then allows for Child Life services to discuss with parents their child’s condition, including what normally calms the patient and what triggers their child may have. With this information, including what maneuvers may help during anesthetic induction, an appropriate plan can be developed, allowing for a more streamlined perioperative experience. Child Life specialists are also very helpful with orienting patients and parents to the perioperative environment as well as teaching them effective coping skills to help them when they are anxious. Importantly, the anesthesiologist may have limited time to interact with the patient and family, and a Child Life specialist can devote more time to creating a bond with the patient and may even be able to stay with them through the induction process.

A recent randomized controlled trial studied the effect of Child Life preparation on anxiety prior to an intravenous induction of anesthesia [50]. Fifty-nine patients aged 3 to 10 years were randomized to a minimum of 15 min with a Child Life specialist or control (no Child Life intervention). There was a significant decrease in anxiety in the intervention group, indicating value in these interactions.

### 3.3. Audiovisual Aids

Portable audiovisual aids such as tablets and smartphones have been used to decrease perioperative anxiety. Cumino and colleagues, in a randomized trial of 84 children aged 4–8 years, found decreased anxiety in those patients that were allowed to play with smartphone applications compared to a control group [51]. Others have found that video-based devices used during induction were at least equivalent to oral premedication with midazolam and PPIA at decreasing anxiety, postoperative pain, and emergence delirium [52,53,54]. Due to ease of use, even for young patients, these distraction techniques have been helpful in assisting with a smooth anesthetic induction. Interestingly, the combination of video distraction and midazolam premedication has not been shown to be better than either of these interventions alone [55].

Virtual reality (VR) devices have also been shown to be effective in anxiolysis. Eijlers and colleagues performed a systematic review and meta-analysis of the use of VR in pediatric patients as a distraction technique during venous access, dental, burn, or oncological care, or prior to general anesthesia. They found that VR was an effective intervention for both pain and anxiety and this held true when observed by both caregivers and professionals [56]. Another study used an immersive VR program in which children could tour the operating room and learn about the perioperative experience. The VR group demonstrated significantly reduced preoperative anxiety but no difference in the incidence or severity of emergence delirium [57]. There are a limited number of studies looking at the use of VR, and advancements in this technology continue. Future study of their use in the medical setting will be important.

### 3.4. Parental Presence during Induction of Anesthesia (PPIA)

PPIA, in which the child and parent do not need to be separated before the child is asleep, has been used in institutions to try to alleviate anxiety during anesthetic induction as well as to promote family-centered care [2]. However, multiple studies have not demonstrated a decrease in anxiety or increased cooperation of the child, especially when compared to premedication [58,59,60]. Importantly, an increase in parental satisfaction has been observed [61,62], and many parents feel that their presence helps their child.

Multiple studies have demonstrated that parental anxiety can actually have a negative effect, with children of anxious parents experiencing more anxiety than children of calm parents [1,63]. Furthermore, persistent behavioral problems up to six months after surgery are 3.2 times more likely in a child with anxious parents compared to calm parents. When both the child and parent were calm, there was no benefit to PPIA [1]. In light of this, an assessment of family members’ levels of anxiety and coping styles is helpful in addition to the evaluation of the patient since this can negatively affect the child.

Appropriate preparation of a parent to reduce anxiety has been shown to improve the effects of PPIA, including training them in distraction techniques and giving them audiovisual aids [64,65]. However, there is one case report that describes a rather unique occurrence during induction of a child with the parent present. Despite reviewing in detail the sequence of events for induction of anesthesia and expectations with the mother, after the child was asleep, the mother picked up her child, disconnected him from monitors, began to shake him vigorously to wake him up, and attempted to leave the induction room with her child. The mother was eventually convinced to put her child back down on the stretcher. The child was allowed to emerge from anesthesia safely and the surgery was ultimately cancelled [66]. This example highlights some of the safely concerns, medico-legal implications, and the importance of proper parental selection for presence at induction.

### 3.5. Premedication

Multiple studies have demonstrated that use of anxiolytic premedication facilitates induction of anesthesia, allowing for a calmer and more controlled experience for the patient [58,67,68,69]. Numerous medication options exist and are most often given orally or transmucosally. In extremely uncooperative patients, IM injection of a sedative medication may be needed.

#### 3.5.1. Midazolam

The premedication most commonly used is the short-acting benzodiazepine midazolam [64]. Oral dosing ranges from 0.25–1 mg/kg and results in a calm, drowsy state in approximate 15–20 min without producing respiratory compromise in otherwise healthy children [70]. However, paradoxical reactions, in which the child becomes agitated and disinhibited, can also occur [71]. Oral midazolam has a bitter taste, which can make administration more difficult in an uncooperative child. In this scenario, midazolam can also be administered IM (0.1 mg/kg), nasally (0.2 mg/kg), or rectally (0.5–1 mg/kg).

In addition to anxiolysis, midazolam also causes anterograde amnesia. Both of these mechanisms are thought to decrease the incidence of negative behaviors after surgery, including separation anxiety and postoperative eating disturbances [72]. While some studies have demonstrated a delay in recovery from anesthesia after the use of oral midazolam premedication, others have not shown this to occur [73].

#### 3.5.2. Ketamine

Ketamine can be given via multiple routes, but most commonly it is administered as either an IM injection or orally as a premedication. For combative, uncooperative patients, IM ketamine (2–5 mg/kg) can be injected undiluted and has an onset time of approximately 10 min. For oral administration, ketamine (3–4 mg/kg) is commonly combined with oral midazolam (0.3 mg/kg) [2]. Common side effects need to be taken into consideration and include increased secretions, nausea, vomiting, psychological disturbances, and prolonged recovery.

#### 3.5.3. Dexmedetomidine

Dexmedetomidine is an alpha-2-adrenergic receptor agonist that has gained popularity as a premedication. Intranasal (IN) dexmedetomidine 1 mcg/kg, when compared to oral midazolam 0.5 mg/kg, was found to provide more effective preoperative sedation and anxiolysis [74]. As opposed to IN midazolam, it does not cause an unpleasant burning sensation. However, it does have a prolonged onset of sedation of up to 30 min, which needs to be taken into account in order to appropriately time its administration. Additionally, dexmedetomidine reduces the volatile anesthetic requirement, decreases the incidence of emergence delirium, and has analgesic properties that decrease the amount of postoperative analgesic medication that is needed [75,76].

### 3.6. Emergence Delirium

Emergence delirium is a dissociated state of consciousness that occurs in the immediate post-anesthesia period and is characterized by non-purposeful movement, thrashing, restlessness and inconsolable crying [77]. Typically, it begins as the child awakens from anesthesia and lasts on average approximately 14 min but can be up to 45 min [78]. Depending on the tools used to measure emergence delirium, the incidence has been reported to range from 5.3% to 80% [2,79]. While it will resolve spontaneously, it is a cause of concern since it can cause injury to the child or loss of the IV catheter as well as prolonging recovery room stay and decrease parent satisfaction [2].

Many risk factors for emergence delirium have been identified. Younger aged children between 2 and 5 years have the highest incidence [80]. While a definitive relationship between emergence delirium and preoperative anxiety has not been proven, the odds of experiencing emergence delirium increases 10% for each increment of 10 points in a child’s preoperative anxiety score (mYPAS) [46]. Both inhalational and intravenous anesthetic agents have been implicated as causative agents of emergence delirium. However, the incidence is higher with the less soluble inhalational agents sevoflurane and desflurane [81]. Finally, postoperative pain may also be a risk factor in young children, with a decreased incidence seen in children administered IN fentanyl intraoperatively [82]. When children underwent pain-free procedures (such as MRI) under sevoflurane anesthesia, there was a significant decrease in emergence delirium when fentanyl 1 mcg/kg IV was administered, suggesting that fentanyl’s sedative effects may be beneficial in reducing its incidence [83].

Multiple assessment tools have been used with varying success to diagnose emergence delirium since it is often difficult to differentiate this from pain in small children. The validated PAED scale consists of five items to help better make this diagnosis in small children and has high sensitivity, specificity, and interobserver reliability [84]. These items, scored on a scale of 0 to 4, include: the child makes eye contact with the caregiver; purposeful movement; awareness of surroundings; restlessness; and whether the child is inconsolable. If the total score is greater than 10, emergence delirium is present.

Multiple pharmacologic and non-pharmacologic methods have been used to try to prevent and treat emergence delirium, with the results of large meta-analyses helping to determine their effectiveness. Opioids, most commonly fentanyl (1 mcg/kg), midazolam, ketamine, and dexmedetomidine (0.3–0.5 mcg/kg), have all been shown to decrease the incidence of emergence delirium. Propofol, administered as a single bolus (1 mg/kg) prior to emergence or given as an infusion during the anesthetic, has also been effective. However, the use of preoperative midazolam premedication and PPIA has not been shown to decrease its occurrence in these meta-analyses [85,86,87]. Ultimately, emergence delirium is self-limited, and, if left untreated, has not been associated with long-term sequelae.

## 4. Conclusions

Perioperative anxiety and fear are common in children presenting for surgery. These emotions can be amplified in patients with known behavioral and emotional disorders, requiring a tailored and well-thought out pre- and postoperative plan to help alleviate the patients’ and parents’ concerns during this vulnerable period. While this review has discussed some of the behavior disorders that children undergoing surgery may present with, others certainly exist. Furthermore, other perioperative challenges, including cultural and linguistic diversity, adverse childhood events, and nuances of the care of very young children in this setting are beyond the scope of this review. Many of these topics require further study to help guide future care. Fortunately, multiple resources and techniques are available to help facilitate a smooth course for many of these patients in order to provide them with the best perianesthetic care possible.

## Figures and Tables

**Table 1:** Overview of common pediatric behavioral and emotional disorders.

Behavioral Disorder	Prevalence (%)	Characterized by	Potential Perioperative Issues
Anxiety	6–13	excessive and uncontrollable worry	separation anxiety extreme fear about specific situation may refuse to communicate or interact panic attacks may complain of abdominal pain, chest pain, headache, dizziness, nausea, or vomiting
Depression	3	persistent feelings of sadness irritability loss of interest in activities feelings of hopelessness thoughts of suicide changes in sleep and eating habits have difficulty concentrating	suicidal risk self-injury or self-destructive behavior substance abuse may complain of headache or abdominal pain may refuse to communicate or interact
Oppositional Defiant Disorder	2–16	angry and irritable mood argumentative and defiant behavior vindictiveness	lack of cooperation/assent violent tantrums aggression towards people substance abuse
Conduct Disorder	6–16 (boys) 2–9 (girls)	aggression towards people and animals destruction of property deceitfulness, lying, or stealing serious violation of rules	lack of cooperation/assent aggression and violence towards people substance abuse
Attention Deficit Hyperactivity Disorder	2–20	inattention impulsivity hyperactivity	preoperative anxiety agitation during induction and emergence postoperative behavioral changes drug interactions (ex: methylphenidate)
Obsessive Compulsive Disorder	1–3	anxiety-provoking thoughts (obsessions) repeated time-consuming behaviors (compulsions)	major anxiety and fear exacerbation of symptoms may require repeated explanations
Post-Traumatic Stress Disorder	1–6 (boys) 3–15 (girls)	intrusive thoughts or memories of the event avoidance of any reminders of the event negative thinking or mood since the event occurred lasting feelings of anxiety or physical reactions	distrust of people separation anxiety refusing to communicate or interact substance abuse suicidal thoughts increased aggression and irritability
Autism Spectrum Disorder	1–2	functional impairment in social communication restricted interests repetitive behaviors sensory processing issues	perioperative anxiety and agitation self-injury and aggression drug interactions (ex: risperidone)
